# Comparative Study of the Effects of Two Dietary Sources of Vitamin D on the Bone Metabolism, Welfare and Birth Progress of Sows Fed Protein- and Phosphorus-Reduced Diets

**DOI:** 10.3390/ani12131678

**Published:** 2022-06-29

**Authors:** Michael Lütke-Dörhoff, Jochen Schulz, Heiner Westendarp, Christian Visscher, Mirja R. Wilkens

**Affiliations:** 1 Institute for Animal Hygiene, Animal Welfare and Farm Animal Behaviour, University of Veterinary Medicine Hannover, Foundation, 30173 Hannover, Germany; jochen.schulz@tiho-hannover.de; 2 Faculty of Agricultural Sciences and Landscape Architecture, University of Applied Sciences Osnabrück, 49090 Osnabrück, Germany; h.westendarp@hs-osnabrueck.de; 3 Institute for Animal Nutrition, University of Veterinary Medicine Hannover, Foundation, 30173 Hannover, Germany; christian.visscher@tiho-hannover.de; 4 Institute of Animal Nutrition, Nutrition Diseases and Dietetics, Faculty of Veterinary Medicine, University of Leipzig, 04103 Leipzig, Germany

**Keywords:** vitamin D, 25-hydroxycholecalciferol, welfare, bone metabolism

## Abstract

**Simple Summary:**

The dietary supply of vitamin D_3_, which is carried out in indoor husbandry due to limited sunlight, often results in a vitamin D status that is lower than that observed in outdoor pig farming. Studies have shown that the use of the vitamin D metabolite 25-hydroxycholecalciferol leads to a more efficient increase in vitamin D status. However, when nutrient intake is adequate, the replacement of the vitamin D source appears to have no or only marginal effects on the performance and bone metabolism of sows. The objective of this study was to investigate the influence of both forms of vitamin D, vitamin D_3_ and 25-hydroxycholecalciferol, on the mineral homeostasis, bone metabolism, welfare and birth progress of sows fed protein- and phosphorus-reduced diets. Supplementation with 25-hydroxycholecalciferol instead of vitamin D_3_ improved vitamin D status and reduced gait changes. However, mineral homeostasis and bone metabolism were not affected by the replacement of the vitamin D form. In conclusion, under conditions of a dietary reduction in protein and phosphorus, the replacement of the vitamin D form seems to help to improve the locomotion of sows, although bone mineralization is probably not affected.

**Abstract:**

To investigate the influence of two dietary sources of vitamin D on the vitamin D status, bone metabolism, welfare and birth progress of gestating and lactating sows, forty-nine multiparous sows were randomly assigned to one of two diets: “CON” (*n* = 25; 50 μg vitamin D_3_/kg feed) and “HYD” (*n* = 24; 50 μg 25-hydroxycholecalciferol/kg feed). The basal diets were protein- and phosphorus-reduced. The trial started on day 3 ante insemination of the sows and ended with weaning of the piglets on day 28 postpartum. Dietary supplementation of 25-hydroxycholecalciferol resulted in improved maternal vitamin D status (*p* < 0.001), fewer gait changes (*p* < 0.01) and longer standing time after feeding (day 5 ante partum; *p* < 0.05) compared to vitamin D_3_. However, the bone markers CrossLaps and osteocalcin were not affected. Overall, the present results suggest that sows fed 25-hydroxycholecalciferol instead of vitamin D_3_ showed improved locomotion and stance strength. However, this outcome is probably not related to altered bone metabolism. The underlying mechanisms must be investigated in further studies.

## 1. Introduction

Sows kept outdoors show a higher vitamin D status than those kept indoors [1]. Dietary supplementation of vitamin D_3_ is considered one of the most widely applied approaches to improve vitamin D status [2]. Vitamin D_3_ is converted via two hydroxylation steps, first to the circulating form 25-hydroxycholecalciferol (25-OHD_3_) in the liver and then to the vitamin D hormone 1,25-dihydroxycholecalciferol (1,25(OH)_2_D_3_) in the kidney [3,4]. In monogastric livestock, dietary vitamin D_3_ can be partially or completely replaced by 25-OHD_3_ [5]. Higher serum concentrations of 25-OHD_3_ have been demonstrated when animals are supplemented with 25-OHD_3_ instead of the equivalent use of vitamin D_3_ [6], probably due to a more efficient intestinal absorption [7].

Despite increased plasma concentrations of 25-OHD_3_, the results of previous studies in sows and their offspring [8,9,10] that investigated the use of 25-OHD_3_ instead of vitamin D_3_ under conditions of adequate protein and phosphorus supply are inconsistent. However, with a dietary reduction in phosphorus, positive effects of 25-OHD_3_ on the performance and digestibility of calcium (Ca) and phosphorus have already been demonstrated in growing pigs [11]. Similar effects under the nutrient-reduced feeding of sows with regard to reproductive and lactation performance are also conceivable. Studies and reviews in humans and rodents indicate that vitamin D has important functions in Ca absorption [12] and bone mineralization [4] as well as in muscle function [13,14]. Therefore, the prevalence of gait changes [14] and compromised birth progress [15] could potentially be positively influenced by a more efficient source of vitamin D.

Based on current knowledge and previous experiments, the following hypotheses were developed. The substitution of dietary vitamin D_3_ with 25-OHD_3_ in sows under conditions of protein- and phosphorus-reduced feeding leads to:

(A) A positive effect on vitamin D status.
(B) Improved Ca and phosphate (P_i_) homeostasis.
(C) A positive influence on bone metabolism.
(D) A lower prevalence of gait changes.
(E) A positive influence on birth progress.

## 2. Materials and Methods

The feeding trial was conducted in accordance with the German animal welfare legislation and was registered with the Lower Saxony State Office for Consumer Protection and Food Safety (Niedersächsisches Landesamt für Verbraucherschutz und Lebensmittelsicherheit, LAVES) under file number 33.8-42502-05-20A504.

### 2.1. Animals and Diets

Forty-nine multiparous sows (Topigs 20, Topigs Norsvin GmbH, Senden, Germany) were randomly allocated to two groups, with 25 sows in the control group and 24 sows in the experimental group on day (d) 3 ante insemination (ai) based on their parity (3.4 to 3.3), body weight (BW) (219.1 to 217.5 kg), body condition score (BCS) (2.9 to 2.9) and backfat thickness (BF) (13.4 to 13.2). While the control group (CON) was supplemented with 50 μg/kg feed vitamin D_3_, the experimental group (HYD) instead received 25-OHD_3_ at a dose of 50 μg/kg diet as a complete replacement throughout gestation and lactation. The gestation diet was fed from the start of the experiment on 3 d ai until 3 d postpartum (pp). Thereafter, the lactation diet was allocated until weaning of the piglets at 28 d pp. The sows selected for the experiment were artificially inseminated with semen from five boars of the Pietráin breed. Care was taken to distribute the boars evenly among the groups to minimize the genetic influence of the sire on the progeny. The semen was purchased from the Erzeugergemeinschaft und Züchtervereinigung für Zucht- und Hybridzuchtschweine in Bayern w.V. (boar station Huntemühlen). The housing conditions (air space, climate, animal density and floor) were the same for both groups. The sows were kept on slatted floors and had constant access to manipulable material in the form of straw and cotton ropes.

The composition of the basic diets in terms of ingredients is shown in Table 1, and the analyzed nutrient contents are shown in Table 2. Table 3 presents the analyzed feed concentrations of vitamin D_3_ and 25-OHD_3_. The vitamin D_3_ and 25-OHD_3_ (Rovimix^®^ Hy-D^®^ 1.25%) used was provided by DSM Nutritional Products GmbH.

### 2.2. Measurements

**Figure 1:**
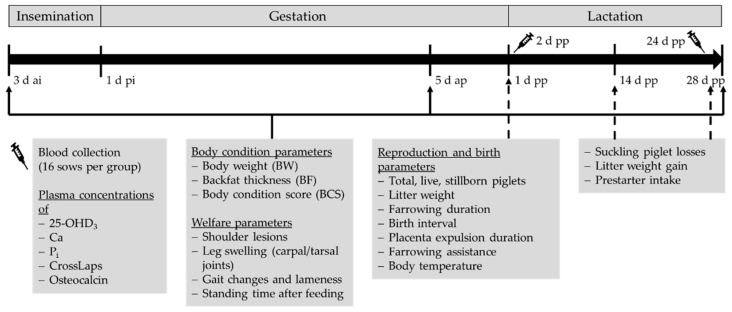
Schematic representation of data collection during the experimental period (3 d ai to 28 d pp; not to scale).

Figure 1 shows a graphical depiction of the experimental procedure.

#### 2.2.1. Sampling and Laboratory Testing

Samples were taken from each of the four feeds, sealed airtight in freezer bags, labeled and frozen at −18 °C until analysis. Proximate analysis of the feed was carried out in the laboratory of the University of Applied Sciences Osnabrück (Weende analysis according to VDLUFA recommendations, method book III, [17]). The Ca contents of the feeds could be determined by means of a flame photometric determination after ashing and absorption in acid. For the determination of P_i_, photometric determination was performed, also after ashing and absorption in acid.

Blood samples were taken from the external jugular vein of a total of 32 sows (16 per diet) on 2 and 24 d pp. Conventional heparin monovettes (9 mL) were used for this purpose. Immediately after blood collection, samples were centrifuged (SBS-LZ-4000/20-12) for ten minutes at 1500 g. The resulting plasma from a single monovette was pipetted into three Eppendorf tubes with lids with a volume of 1.5 mL each and frozen at −18 °C until analysis. In the Institute of Physiology and Cell Biology of the University of Veterinary Medicine Hanover Foundation, the Ca and P_i_ concentrations from the lithium-heparinized plasma samples were determined by standard spectrometric analyses using the o-cresolphthalein complexone and the vanadate-molybdate method. Analysis of the bone resorption marker CrossLaps and the bone formation marker osteocalcin was performed using the Serum CrossLaps^®^ ELISA (Immundiagnostic System GmbH, cat.-no. AC-2F1, Frankfurt am Main, Germany) and the MicroVue Osteocalcin EIA Kit (Quidel Corp., cat.-no. 8002, San Diego, CA, USA) according to the manufacturer´s instructions. Intra- and inter-assay coefficients of variance in our laboratory were 9.4% and 3.5% for CrossLaps and 4.4% and 4.5% for osteocalcin. Both assays haven been used successfully for porcine samples before.

The plasma and feed concentrations of 25-OHD_3_ were determined at the DSM Nutritional Products Analytical Research Centre (Kaiseraugst, Switzerland) using high-pressure liquid chromatography–mass spectrometry.

#### 2.2.2. Feed Intake and Body Condition Development

The gestating sows were kept in group housing in two separate pens and were thus fed via two separate stations (Mannebeck INTEC 6001) and the associated bag containers. This made it possible to separate the feeding of the CON and HYD groups. With the help of transponders in the ear, each sow could be fed with the individually allocated amount of feed (mean, 3.2 kg). Animal-specific feed residues could not be recorded during gestation. Five days before the calculated mean birth date (5 d ante partum (ap)), the sows were housed in the farrowing pens provided for the experiment and continued to be fed separately with the appropriate feed (CON/HYD). The feed was allocated manually in the farrowing pen using previously calibrated measuring cups. The automatic feeding system that is usually used on the farm was switched off for the duration of the trial. Each sow received a feed card documenting the daily feed quantities and feed residues. The feeding of the prestarter for the suckling piglets was also carried out per litter with the aid of calibrated cups and a separate feed card, which served to document the amounts given and the residues. The adjustment of the feed quantity for the sows and the prestarter quantity for the suckling piglets (reduction, increase) was carried out according to the feed residues.

The condition parameters included BW, BF and BCS, recorded at start (3 d ai), transfer to farrowing pen (5 d ap) and weaning (28 d pp). A mobile single animal scale (Texas-Trading FX1 weighing system) with a weighing range of up to 2.000 kg and a measurement accuracy of ±0.5% of the displayed weight was used to determine the BW of the sows. The BCS was scored according to a 5-level scoring system devised by Li et al. [18]. Here, 1 = emaciated (backbone and pin bones are visible), 2 = thin (backbone and ribs are easily palpable with pressure), 3 = fit (backbone and ribs are barely palpable with firm pressure), 4 = fat (backbone and ribs are not palpable with pressure) and 5 = very fat (clearly overweight). The BF was measured with an ultrasound (esaote, Tringa Linear). The transducer was placed bilaterally at the level of the last rib 6 cm lateral to the spine (P2 method) [19] and the two fat layers overlying the longissimus dorsi muscle (subcutis and interfacial fat layer), including connective tissue, were measured.

#### 2.2.3. Welfare Parameters

Additional welfare parameters were collected at the same three time points as the body condition scoring. These included shoulder lesions, which were scored as follows: 0 = no evidence of a shoulder lesion or redness; 1 = redness without penetration into the tissue (no skin breakdown); and 2 = clear visible open wound or lesion (skin breakdown) (modified after Welfare Quality^®^ [20]). In addition, the legs (carpal/tarsal joints) were examined for swelling [20]. If at least one area of swelling (>2 cm) was visible, a score of 1 was documented; otherwise, a score of 0 was given. A gait score was determined to allow the assessment of gait changes in the sows. For this purpose, the gait of each sow was dynamically rated from the side as well as from the front and back on a 16 m outward and return walk according to the following key (modified after Grégoire et al. [21] and Main et al. [22]): 0 = no gait changes, sow walks with even steps; 1 = gait changes, abnormal step length, movement not fluid (asymmetrical walking); 2 = mild lameness, step is shortened, at least one leg is only placed on the ground with a minimum of weight; and 3 = severe lameness, the affected limb cannot bear weight, or the sow cannot move under its own power. Grade 3 was not assigned to any sows in the present study. Since the evaluation was conducted in the housing, the animals were used to the floor conditions. Accordingly, the floor was the same for both feeding groups during the evaluation and the entire trial period. In addition, the standing time after feeding [21] was measured using a stopwatch on the same dates in connection with the feeding of the sows. All the monitoring and surveys carried out in the present trial were performed by the same person.

#### 2.2.4. Reproduction and Birth Parameters

As the sows’ births were not induced, there was a time period of six days while births were continuously recorded (24 h). This made it possible to measure the duration of birth and the time until the expulsion of the placenta as well as the interval between piglets. Necessary birth assistance and the total number of piglets born, alive, dead and mummified, were also documented. Birth assistance was provided if no new piglet or placenta was expelled after a 60-minute waiting period and the sow showed recognizable contractions or when another piglet was suspected. In the time period of 12 to 24 h after the complete expulsion of the placenta, the newborn piglets were individually weighed using a platform scale (Kern EOB 35K10, measuring range: max. 35 kg, measuring accuracy of 0.01 kg). Records were also kept for individual stillborn piglets, and their weights were documented. In order to achieve litter numbers that were as homogeneous as possible, litter balancing was carried out after weighing in the time window 12–36 h after birth, taking into account sows’ previous performance and litter numbers. Care was taken to ensure that the piglets were only moved within the respective feeding groups of the mother sows. Excess piglets were housed with nurse sows and their weight documented. They were not included in further evaluation from this point onward. At the middle of the lactation period (14 d postnatum (pn)) (Kern EOB 35K10, measuring range: max. 35 kg, accuracy 0.01 kg) and at weaning (28 d pn) (Sartorius EB60FEG-I, measuring range: 60 kg, accuracy 0.002 kg), the weight of each individual piglet was again determined. All suckling piglet losses were weighed and noted. In the first three days after birth, rectal body temperature was measured in all sows after morning feeding and documented. This allowed postpartum temperature to be assessed according to a key based on values from Papadopoulos et al. [23] and Kemper [24], where 0 = <39.6 °C; 1 = 39.6–39.9 °C; 2 = ≥40.0 °C. The corresponding score was given even if the temperature range was exceeded only once.

### 2.3. Statistical Analysis

Statistical data analyses were performed using the SAS^®^ Enterprise Guide^®^ statistical analysis system (version 7.1, SAS Institute Inc. Cary, NC, USA). Data were tested for normal distribution, and the mean and standard deviation (SD) for each parameter were determined. A comparison of the groups was performed using analysis of variance (ANOVA) or the nonparametric two-sample Wilcoxon test. Depending on the amount of available data, either Pearson’s chi-square homogeneity test or Fisher’s test was used to analyze the qualitative parameters. Plasma data were analyzed by Repeated Measures ANOVA (time or time and group) followed by Holm–Sidak´s test to reveal significant group differences using GraphPad Prism 9.1.2 (GraphPad Software, San Diego, CA, USA); no samples were excluded from the data. When *p* < 0.05, the null hypothesis of no significant differences between the control and experimental groups was rejected.

## 3. Results

### 3.1. Plasma Concentrations of 25-OHD_3_, Ca, P_i_, CrossLaps and Osteocalcin

Plasma concentrations of 25-OHD_3_, Ca, P_i_, CrossLaps and osteocalcin are shown in Table 4. Sows fed diets containing 25-OHD_3_ (50 μg/kg) instead of vitamin D_3_ (50 μg/kg) had higher plasma concentrations of 25-OHD_3_ after farrowing (2 d pp) and a few days before weaning (24 d pp) (*p* < 0.001). It can be seen for Ca and P_i_ as well as for the bone markers osteocalcin and CrossLaps that there is no significant difference in relation to the vitamin D form. 

### 3.2. Feed Intake and Body Condition Development

The average daily feed intake (ADFI) of sows was the same between groups during lactation. The influence of the vitamin D form on the condition parameters BW, BF and BCS is shown in Table 5. All these parameters significantly changed during the observation period (*p* < 0.001). In terms of BF, the control group showed a higher increase (+25%) during gestation than the experimental group. However, BF loss during lactation was also higher in the control group (+31%). There were no differences in BW and the BCS between the feeding groups within the trial.

### 3.3. Welfare Parameters

**Figure 2:**
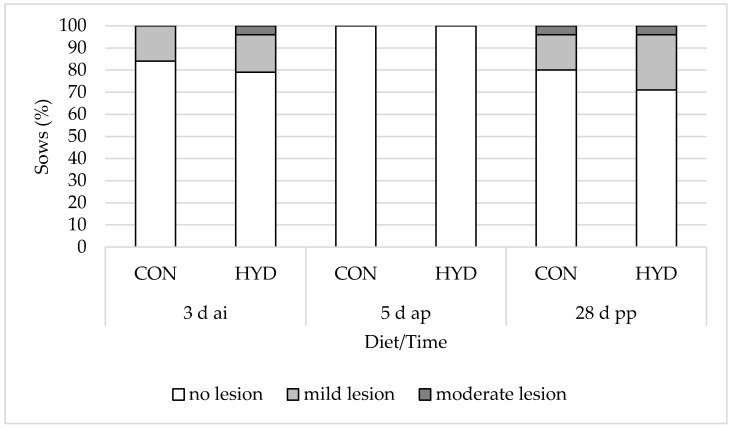
Effect of dietary vitamin D source on prevalence (%) of shoulder lesions of sows. CON, 50 µg/kg vitamin D_3_ (*n* = 25); HYD, 50 µg/kg 25-OHD_3_ (*n* = 24).

**Figure 3:**
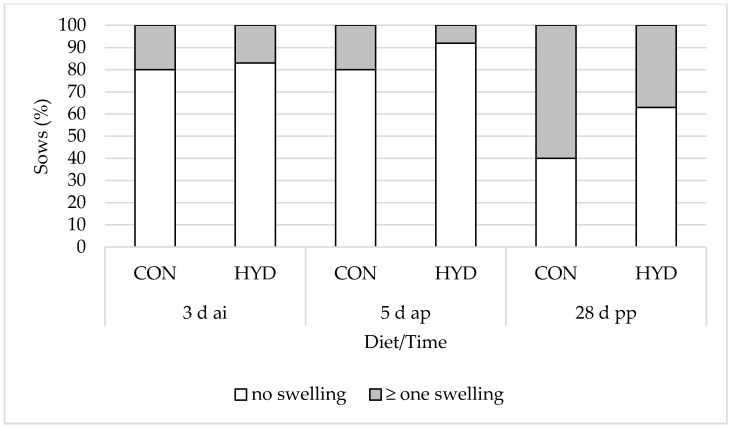
Effect of dietary vitamin D source on prevalence (%) of leg swelling (carpal/tarsal joints) of sows. CON, 50 µg/kg vitamin D_3_ (*n* = 25); HYD, 50 µg/kg 25-OHD_3_ (*n* = 24).

**Figure 4:**
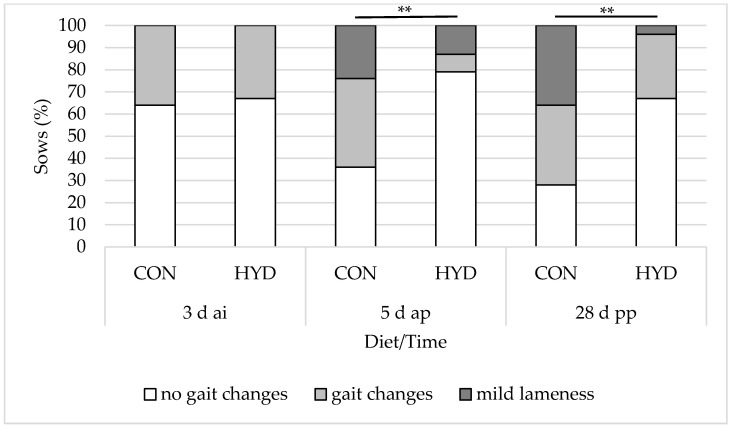
Effect of dietary vitamin D source on prevalence (%) of gait changes and mild lameness of sows. CON, 50 µg/kg vitamin D_3_ (*n* = 25); HYD, 50 µg/kg 25-OHD_3_ (*n* = 24). ** *p* < 0.01 at respective time points.

Figure 2, Figure 3 and Figure 4 show the influence of dietary vitamin D form on the welfare parameters of shoulder lesions, leg swelling (carpal/tarsal joints) and gait changes. There were no significant differences in shoulder lesions and leg swelling (carpal/tarsal joints) between the feeding groups. While 79% of the sows in the HYD group showed no signs of gait changes and mild lameness 5 d ap, this was the case for only 36% of the sows in the CON group. At weaning, 67% of the experimental sows and only 28% of the control sows showed no signs of gait changes and mild lameness. Overall, the effects were highly significant at both 5 d ap (*p* = 0.007) and 28 d pp (*p* = 0.006). None of the sows in the study showed severe lameness. 

Table 6 presents the effects of dietary vitamin D form on standing time after feeding. At 5 d ap, sows in the HYD group stood on their legs 6 min longer than sows in the CON group (*p* = 0.03). No differences were detected at the start and at weaning.

### 3.4. Reproduction and Birth Parameters

The influence of the vitamin D form on the reproduction parameters is shown in Table 7. The two groups do not differ from each other in the number of total and live born piglets nor in the number of stillborn piglets. However, the number of weaned piglets was higher in the control group, with 14.0 piglets, than in the experimental group, with 13.0 piglets. The suckling piglet losses were 17.0% (CON) and 21.8% (HYD) and did not differ significantly. Litter weight, litter weight gains and within-litter weight variance were at a similar level at each measurement time point. Similarly, there was no effect on the supplemental feed intake of the suckling piglets. 

The measurement of the parameters of birth progress and postnatal rectal body temperature are shown in Table 8. The total duration of farrowing was 288 ± 112 min (until the last piglet, mean ± SD) and 517 ± 260 min (including placental expulsion) in the CON group and 334 ± 173 min and 480 ± 186 min (including placental expulsion) in the HYD group. Accordingly, placental expulsion was 228 ± 221 min in the CON group and 147 ± 94 min in the HYD group, tending to be shorter. The average piglet birth interval was 16 ± 6 min in the CON group and 19 ± 11 min in the HYD group. In percentage terms, 22% of the sows in the CON group required obstetric assistance at least once, while in the HYD group, it was only 9%. Body temperature was similar in the first three days after birth in both groups. 

## 4. Discussion

### 4.1. Vitamin D Status, Plasma Minerals and Bone Metabolism

In the present study, vitamin D status was significantly increased by supplementing 25-OHD_3_ instead of vitamin D_3_. At the end of gestation and shortly before weaning, plasma concentrations of 25-OHD_3_ were approximately increased by 90% in HYD sows in comparison to the CON group (46.5 ng/mL vs. 24.5 ng/mL and 73.2 ng/mL vs. 37.7 ng/mL) resulting in levels comparable to those detected in sows kept in outdoor housing systems (57 ng/mL, [1]; 67 ng/mL, [25]). In contrast, the results of the CON animals indicate that at least individual sows presented with 25-OHD_3_ plasma concentrations below the reported reference range of 35–70 ng/mL [26]. In line with the present results, Flohr et al. [8], Thayer et al. [9], Weber et al. [10] and Coffey et al. [27] also demonstrated positive effects of replacing vitamin D_3_ with 25-OHD_3_ on the vitamin D status of sows. 

In humans, an inverse relationship has been demonstrated between plasma concentrations of 25-OHD_3_ and intestinal Ca absorption, as well as bone mineral density and fall risk, especially when plasma concentrations of 25-OHD_3_ are below 32 ng/mL [28]. Bouillon and Rosen [29] confirmed that Ca absorption and bone mineral density are impaired at low serum concentrations of 25-OHD_3_ (<20 ng/mL) in humans. Adequate Ca levels are required to ensure neuromuscular function and optimal bone mineralization [30].

In sows, mineral homeostasis is challenged by gestation and lactation [31]. As lameness is one of the most important health issues in modern pig production [32], our rationale to conduct this study was to improve bone mineralization by increasing vitamin D status. However, supplementing 25-OHD_3_ instead of vitamin D_3_ did not exert any effects on plasma Ca, phosphate, the bone resorption marker CrossLaps or the bone formation marker osteocalcin. All these parameters were influenced by sampling time, indicating the effects of lactation, the change in mineral demand and the dynamics in bone metabolism and mineral absorption, but our hypothesis that 25-OHD_3_ would have beneficial effects on mineral homeostasis and bone metabolism can be rejected. 

### 4.2. Body Condition and Composition

Studies conducted in humans have shown that there is a correlation between low vitamin D status and increased body fat percentages [33,34]. Furthermore, it was shown in fattening broiler chicks that a supplementation with 25-OHD_3_ accompanied by higher plasma concentrations of 25-OHD_3_ stimulates breast muscle development and increases protein deposition, probably via the mTOR pathway [35,36]. In the present study, dietary supplementation with 25-OHD_3_ had no effect on daily feed intake in lactation and the change in body weight during gestation and lactation. This is in line with other studies [8,9]. However, HYD sows showed a less pronounced increase in back fat thickness during gestation and a lower loss of back fat thickness during lactation despite unaffected weight gain. From this observation, it might be concluded that the treatment affects the body composition of sows. Due to the limited number of animals in the present study, this assumption should be verified in further studies using more sophisticated techniques to quantify muscle tissue. In contrast to the present results, Flohr et al [8] did not demonstrate an effect on back fat thickness. In the broiler chicks, the impact of 25-OHD_3_ supplementation on muscle development was verified by a very elegant estimation of in vivo protein synthesis using an injection of ^15^N-labelled phenylalanine combined with in vitro studies using quail myoblast cells [36]. It might be speculated that under a marginal dietary protein supply these mechanisms gain importance. Flohr et al. [8] fed much a higher crude protein content (14.8% to 15.2% during gestation, 19.5% to 20.1% during lactation) in comparison to the diet used in the present study (13.3% to 13.6% during gestation, 15.6% to 15.8% during lactation). 

### 4.3. Musculoskeletal System

Shoulder lesions represent a welfare problem and affect 5% to 50% of all sows worldwide [37]. In the present experiment, shoulder lesions were recorded at the beginning (insemination, CON, 16% vs. HYD, 21%) and at the end (weaning, CON, 20% vs. HYD, 29%) of the experiment. These numbers are in agreement with data published by Friedrich et al. [38] (72% to 88% without lesions). Differences between the diets could not be demonstrated. At weaning, 60% of the CON sows and 37% of the HYD sows presented with leg swellings (carpal/tarsal joints). This increase can be explained by more time spent lying and less individual space during lactation, which is also demonstrated by the shorter interval between feeding and lying down 5 d ap and 28 d pp (Table 6).

The prevalence of lameness in sows depends on factors associated with management, housing and diet and can vary substantially [39,40]. In the present study, the prevalence of gait changes (asymmetrical gait or abnormal stride length) and mild lameness increased in both groups during the experimental period. However, we observed significant differences between the two feeding groups. Before parturition, gait changes or mild lameness was observed in 64% of the CON sows but only in 21% of the HYD animals. At weaning, the prevalence amounted to 72% in the CON group but only 33% in the HYD group. Despite the high level of agreement between two different independent observers (94%), the method of gait assessment is still subjective and strongly depends on the experience of the observer [22]. Therefore, we additionally performed the more objective method of recording standing time after feeding. Grégoire et al. [21] showed that lame sows spend less time in a standing position after feeding than non-lame sows. In accordance with our gait assessment, the interval between feeding and laying-down decreased from 50.9 min (CON) and 44.2 min (HYD) to 22.7 min (CON) and 29.0 min (HYD) around parturition and to 17.6 min (CON) and 19.0 min (HYD) at weaning. At 5 d ap, the difference between the groups was significant. 

Against the background of the data on body composition, our results on gait changes and time spent standing might indicate that the supplementation with 25-OHD_3_ resulted in more muscle mass and strength and thus less gait changes and less chance for injuries and lesions. Ceglia et al. [41] performed a randomized, double-bind, placebo-controlled study in elderly women who were supplemented with 4000 IU (100 µg) vitamin D_3_ daily. After four months, serum concentrations of 25-OHD_3_ had increased from 17 ng/mL to 32 ng/mL and the expression of the vitamin D receptor in cells obtained by muscle biopsies was increased in the treatment group [41]. However, no effects could be revealed on the functional level. In contrast, vitamin D was shown to support the healing of muscle tissue in rats subjected to crush injury and in humans experiencing exercise-induced muscle injury [42,43]. Given that the rapid changes between anabolism and catabolism in modern, high performing sows kept on a restricted protein supply include a periodic process of regeneration, different aspects regarding the mode of action that are currently under discussion have been reviewed recently by Iolascon et al. [44]. 

### 4.4. Reproductive Traits

According to Merhi et al. [45], vitamin D deficiency is associated with a lower ovarian reserve in humans. Therefore, it might be speculated that increasing the serum 25-OHD_3_ concentrations to the level found in outdoor housing systems by administering 25-OHD_3_ instead of vitamin D_3_ might improve fertility in sows. However, Flohr et al. [8], Weber et al. [10] and Thayer et al. [9] could not demonstrate a positive effect of 25-OHD_3_ on the number of piglets born. Positive effects were only shown in one study, in which the experimental feeding was started 43 d before breeding [27]. Gilts supplemented with a combination of 12.5 μg/kg vitamin D_3_ and 50 μg/kg 25-OHD_3_ instead of 62.5 μg/kg vitamin D_3_ showed an increase in the number of fetuses (12.7 vs. 10.2) without any differences in individual piglet weight when they were sacrificed on the 90th day of gestation. Due to the study design, it remains open whether this would also have resulted in more piglets born alive or weaned. It also has to be pointed out that selection for more piglets born in the last decades has resulted in lower individual birth weights, higher variation coefficients in birth weight and increased piglet mortality [46]. Therefore, it remains questionable whether an additional increase in the ovulation rate could really be regarded as a positive effect.

Piglet survival as well as the reproductive health of the sow is associated with a fast and uncomplicated birth [47]. In line with former reports [8,9], the respective reproductive traits were not affected by the treatment in the present study except for a tendency towards faster expulsion of fetal membranes (−81 min) in the HYD group. However, even though piglet mortality was not significantly affected, the number of weaned piglets was slightly decreased in the HYD group (13.0 vs. 14.0). While Flohr et al. [8] and Thayer et al. [9] found no effects of the supplemented vitamin D metabolite on the number of weaned piglets and piglet mortality, Weber et al. [10] also demonstrated a lower proportion of weaned piglets relative to live born piglets with 25-OHD_3_ supplementation. In the present study, the reduced number of weaned piglets in the HYD group (13.0 vs. 14.0) could be explained by a slightly higher number of piglets in the CON group after cross-fostering (17.3 vs. 16.9) and perhaps an increased activity of the sows in the HYD group indicated by a longer standing time. However, the suckling piglet losses did not differ.

In humans, low 25-OHD_3_ levels in mothers result in lower birth weights of their offspring [48,49]. Weber et al. [10] showed an increase in individual birth weight per piglet (+8%) and total litter weight (+17%) with supplementation of 25-OHD_3_ instead of vitamin D_3_. As in the studies performed by Flohr et al. [8] and Thayer et al. [9], no alterations of piglet weight was found in our investigation. Since the control group in the study by Weber et al. [10] was characterized by a very low vitamin D status (14.0–22.6 ng/mL), it could be hypothesized that the replacement of vitamin D_3_ with 25-OHD_3_ affects offspring birth weight only when a maternal vitamin D insufficiency is compensated by the treatment. 

## 5. Conclusions

In summary, we confirmed hypothesis A: vitamin D status in sows supplemented with 25-OHD_3_ was improved. However, dietary supplementation with 25-OHD_3_ instead of vitamin D_3_ did not influence mineral homeostasis and bone metabolism as assessed by measuring respective markers, so hypotheses B and C cannot be supported. The most interesting results are related to gait changes: hypothesis D, a reduced prevalence of gait changes, could be confirmed. This observation might be related to more muscle tissue in HYD sows in comparison to CON animals rather than to an impact on bone. Hypothesis E was partially confirmed: although not verified statistically, the necessity for assistance during farrowing was reduced in comparison to the CON group and the HYD sows showed a tendency for faster expulsion of the fetal membranes. Further studies are needed to further investigate whether an improved vitamin D status achieved by UV-exposure or 25-OHD_3_ supplementation can really increase mobility and fitness in high performing sows kept on a diet restricted in protein supply.

## Figures and Tables

**Table 1:** Ingredient composition of the basic diets.

Ingredient (%)	Gestation	Lactation
Barley	40.2	20.0
Wheat bran	21.8	11.0
Wheat	15.0	33.5
Corn	-	10.0
Molasses pulp	4.0	-
Sunflower extraction meal	3.3	-
Sunflower extraction meal (hydrothermal pressure)	3.0	3.0
Sugar beet molasses	3.0	-
Wheat, broken down	2.5	2.5
Calcium carbonate	1.5	1.6
Barley, broken down	1.3	1.3
Corn, broken down	1.3	1.3
Sunflower extraction meal (steam-heated)	1.0	11.2
Sodium chloride	0.7	0.5
Vegetable fatty acids (sunflower, palm, rape kernel)	0.2	1.7
Calcium sodium phosphate	-	0.35
Soybean oil	-	0.50
Palm oil	0.01	0.02
Minor components *	1.19 *	1.53 ^#^

* Feed additives per kg feed in the gestation diet: 12,000 IU vitamin A, 2000 IU vitamin D or 50 μg 25-hydroixycholecalciferol, 120 mg vitamin E, 50 mg carnitine, 120 mg iron, 2 mg iodine, 10 mg copper, 60 mg manganese, 105 mg zinc, 0.35 mg selenium, 5.00 × 10^9^ CFU *Saccharomyces cerevisiae* (NCYC Sc 47), 700 FYT 6-phytase (EC.3.1.3.26), 225 mg clinoptilolite, 830 mg bentonite, ^#^ Feed additives per kg feed in the lactation diet: 12,000 IU vitamin A, 2000 IU vitamin D or 50 μg 25-hydroixycholecalciferol, 80 mg vitamin E, 30 mg carnitine, 120 mg iron, 2 mg iodine, 10 mg copper, 60 mg manganese, 105 mg zinc, 0.35 mg selenium, 5.00 × 10^9^ CFU *Saccharomyces cerevisiae* (NCYC Sc 47), 700 FYT 6-phytase (EC.3.1.3.26), 225 mg clinoptilolite, 175 mg bentonite.

**Table 2:** Chemical composition of the experimental diets.

Parameter	Unit	Gestation		Lactation	
		CON	HYD	CON	HYD
Crude protein	% as-fed basis	13.6	13.3	15.8	15.6
Crude fat		2.6	2.7	4.7	4.4
Crude fiber		6.3	6.6	4.1	3.6
Crude ash		5.4	5.3	5.1	4.7
Starch		36.4	35.6	39.9	41.6
Calcium		0.67	0.66	0.77	0.65
Phosphorus		0.48	0.49	0.47	0.45
Metabolizable energy *	MJ/kg as-fed basis	11.9	11.8	13.3	13.5

* Calculated in accordance with Stangl [16].

**Table 3:** Analyzed mean feed concentrations of vitamin D_3_ and 25-OHD_3_ (μg/kg).

Vitamin D Source	Gestation	Lactation
CON (vitamin D_3_, μg/kg)	45	38
HYD (25-OHD_3_, μg/kg)	43	35

**Table 4:** Effect of dietary vitamin D source on plasma concentrations of 25-OHD_3_, Ca, P_i_, CrossLaps, osteocalcin and osteocalcin/CrossLaps ratio of sows at 2 and 24 d pp; mean ± SD and results of a Repeated Measures ANOVA. Different superscripts within a row indicate significant differences between groups at a certain day revealed by Holm–Sidak´s post-test.

Parameter	CON	HYD	*p*-Value Group	*p*-Value Time	*p*-Value Interaction
25-OHD_3_, ng/mL					
2 d pp	24.5 ^a^ ± 4.9	46.5 ^b^ ± 10.1	<0.001	<0.001	<0.001
24 d pp	37.7 ^a^ ± 7.7	73.2 ^b^ ± 10.5			
Ca, mmol/L					
2 d pp	2.31 ± 0.16	2.29 ± 0.16	0.56	<0.001	0.87
24 d pp	2.52 ± 0.15	2.48 ± 0.21			
P_i_, mmol/L					
2 d pp	2.03 ± 0.22	2.04 ± 0.24	0.11	<0.001	0.11
24 d pp	1.47 ± 0.15	1.68 ± 0.33			
CrossLaps, ng/mL					
2 d pp	0.66 ± 0.24	0.71 ± 0.33	0.41	<0.001	0.83
24 d pp	1.24 ± 0.50	1.33 ± 0.34			
Osteocalcin, ng/mL					
2 d pp	60.9 ± 34.3	70.1 ± 38.3	0.29	<0.001	0.59
24 d pp	109 ± 23.6	130 ± 80.5			
Osteocalcin/CrossLaps, ratio					
2 d pp	100 ± 60.1	129 ± 104	0.38	0.53	0.50
24 d pp	101 ± 39.7	105 ± 76.3			

CON, 50 µg/kg vitamin D_3_ (*n* = 16); HYD, 50 µg/kg 25-OHD_3_ (*n* = 16). ^a,b^ Values with different superscript letters within the same row differ significantly (*p* < 0.05).

**Table 5:** Effect of dietary vitamin D source on condition development of sows (mean ± SD).

Parameter	CON	HYD	*p*-Value
ADFI (gestation), kg ^1^	3.15 ± 0.06	3.16 ± 0.07	0.73
ADFI (lactation), kg	5.74 ± 0.49	5.74 ± 0.45	0.94
BW, kg			
3 d ai	219.1 ± 27.5	217.5 ± 25.2	0.83
5 d ap	292.3 ± 20.2	286.4 ± 20.4	0.10
28 d pp	239.3 ± 24.9	236.5 ± 23.7	0.59
BW gain (gestation), kg	73.2 ± 14.2	68.9 ± 11.8	0.10
BW loss (lactation), kg	53.0 ± 11.1	49.9 ± 8.9	0.20
BW loss (lactation) ^C^, kg	26.4 ± 9.3	24.9 ± 9.7	0.49
BF, mm			
3 d ai	13.4 ± 2.8	13.2 ± 2.9	0.78
5 d ap	18.5 ^a^ ± 3.7	16.9 ^b^ ± 3.6	<0.01
28 d pp	14.2 ± 2.5	14.0 ± 3.3	0.93
BF gain (gestation), mm	5.1 ^a^ ± 1.7	3.8 ^b^ ± 1.3	<0.01
BF loss (lactation), mm	4.2 ^a^ ± 1.9	2.9 ^b^ ± 1.4	<0.01
BCS, Score			
3 d ai	2.9 ± 0.8	2.9 ± 1.0	0.50
5 d ap	3.4 ± 0.5	3.3 ± 0.6	0.30
28 d pp	2.8 ± 0.6	2.9 ± 0.5	0.43
BCS gain (gestation), score	0.5 ± 1.0	0.4 ± 0.9	0.50
BCS loss (lactation), score	0.6 ± 0.7	0.5 ± 0.7	0.41

CON, 50 µg/kg vitamin D_3_ (*n* = 25); HYD, 50 µg/kg 25-OHD_3_ (*n* = 24). ^1^ Amount offered, feed residues could not be measured. ^C^ Correction of born piglets. ^a,b^ Values with different superscript letters within the same row differ significantly (*p* < 0.05).

**Table 6:** Effect of dietary vitamin D source on standing time after feeding (mean ± SD, min).

Parameter	n^1^/n^2^	CON	HYD	*p*-Value
3 d ai	25/24	50.9 ± 69.7	44.2 ± 60.8	0.72
5 d ap	25/23	22.7 ^a^ ± 7.9	29.0 ^b^ ± 11.4	0.03
28 d pp	23/22	17.6 ± 7.6	19.0 ± 7.0	0.55

CON, 50 µg/kg vitamin D_3_; HYD, 50 µg/kg 25-OHD_3_; n^1^, number of CON-sows; n^2^, number of HYD-sows. ^a,b^ Values with different superscript letters within the same row differ significantly (*p* < 0.05). Data missing for animals that were not properly observed (5 d ap: 1 HYD sow, 28 d pp: 2 CON sows and 2 HYD sows).

**Table 7:** Effect of dietary vitamin D source on reproduction parameter of sows (mean ± SD).

Parameter	n^1^/n^2^	CON	HYD	*p*-Value
Number of piglets, n				
Total born	25/22	19.2 ± 3.1	19.3 ± 3.0	0.90
Born alive	25/22	18.0 ± 2.7	17.5 ± 2.6	0.48
Born dead	25/22	1.2 ± 1.5	1.8 ± 1.9	0.19
Born mummified	25/22	0.5 ± 0.7	0.4 ± 1.1	0.68
Stillborn rate (%)	25/22	5.6 ± 6.8	8.9 ± 8.7	0.15
After cross-foster	25/24	17.3 ± 2.7	16.9 ± 1.8	0.54
Middle of lactation period	25/24	14.2 ± 1.5	13.5 ± 1.3	0.06
Weaned	25/24	14.0 ^a^ ± 1.4	13.0 ^b^ ± 1.5	0.03
Suckling piglet losses	25/24	3.3 ± 3.7	3.9 ± 2.6	0.55
Litter weight, kg (WLWV)				
Total born	25/22	26.6 ± 3.9 (0.10)	26.2 ± 3.5 (0.11)	0.49 (0.69)
Born alive	25/22	25.4 ± 3.5 (0.09)	24.1 ± 3.6 (0.10)	0.30 (0.34)
Born dead	25/22	2.0 ± 1.6 (0.15)	2.7 ± 1.8 (0.05)	0.29 (0.07)
After cross-foster	25/24	24.5 ± 3.3 (0.06)	23.0 ± 3.5 (0.08)	0.14 (0.22)
Middle of lactation period, 14 d pp	25/24	50.5 ± 8.3 (0.52)	49.4 ± 9.2 (0.54)	0.67 (0.85)
Weaning, 28 d pp	25/24	95.9 ± 11.3 (1.78)	92.4 ± 14.0 (1.75)	0.34 (0.91)
Litter weight gain, kg				
1–14 d pp ^3^	25/24	26.0 ± 8.4	26.4 ± 8.3	0.88
15–28 d pp	25/24	45.4 ± 5.3	43.0 ± 6.5	0.16
1–28 d pp ^3^	25/24	71.4 ± 11.7	69.4 ± 13.4	0.57
Prestarter intake (g) ^4^	25/24	17.1 ± 7.0	16.7 ± 6.9	0.86

CON, 50 µg/kg vitamin D_3_; HYD, 50 µg/kg 25-OHD_3_. n^1^, number of CON-sows; n^2^, number of HYD-sows; ^3^ After cross-foster; ^4^ 14–28 d pn. WLWV, within-litter weight variance. ^a,b^ Values with different superscript letters within the same row differ significantly (*p* < 0.05). Data on birth are missing for two sows of the HYD group. One animal developed fever shortly before birth which lead to nine dead piglets, another one gave birth to only four piglets and was considered to be an outlier. As the sows recovered quickly, they were used for cross-foster later on.

**Table 8:** Effect of dietary vitamin D source on birth parameters (mean ± SD).

Parameter	CON	HYD	*p*-Value
Farrowing duration, min	288 ± 112	334 ± 173	0.34
Birth interval, min	16 ± 6	19 ± 11	0.28
Placenta expulsion duration, min	228 ± 221	147 ± 94	0.07
Farrowing assistance, %	22	9	0.41
Body temperature (1–3 d pp), °C	39.0 ± 0.3	39.0 ± 0.3	0.63
<39.6 °C, %	65	68	0.50
39.6–39.9 °C, %	13	23	
≥40.0 °C, %	22	9	

CON, 50 µg/kg vitamin D_3_ (*n* = 23); HYD, 50 µg/kg 25-OHD_3_ (*n* = 22). Data missing for four sows. One animal developed fever shortly before birth which lead to nine dead piglets, another one gave birth to only four piglets and was considered to be an outlier. Two sows farrowed shortly before the observer arrived at the barn.

## Data Availability

The data presented in this study are available in this manuscript.
